# Change in Incidence and Severity of Abusive Head Trauma in the Paediatric Age Group Pre- and During COVID-19 Lockdown in the North East of England

**DOI:** 10.22599/bioj.265

**Published:** 2022-08-23

**Authors:** Thomas Salisbury, Neda Qurashi, Qasim Mansoor

**Affiliations:** 1 Northern Deanery, GB; 2 South Tees NHS Trust, GB

**Keywords:** child abuse, abusive head trauma, retinal haemorrhage, COVID-19, non-accidental injury

## Abstract

**Background:**

*Abusive head trauma* (AHT) is currently the accepted terminology that encompasses previously used terms such as non-accidental injury (NAI) or non-accidental head injury (NAHI) and shaken baby syndrome (SBS). It is AHT and its ocular manifestations that ophthalmologists are vital in identifying and reporting.

**Objectives:**

To investigate whether there is a change in the incidence or severity of AHT pre- and during COVID-19 lockdown.

**Participants and Settings:**

AHT cases reported between March–June 2019 and March–June 2020. Data will be collected from ***** **** ********* NHS Foundation Trust.

**Methods:**

A retrospective comparative study.

**Main Outcome Measures:**

- Comparison of total number of children reported to child protection services pre- and during lockdown.
- Severity of reported cases.
- Ophthalmic involvement.

**Results:**

Of the pre-lockdown safeguarding referrals, 5/61 (8.19%) had confirmed AHT, and 4/40 (10%) of the during lockdown group were confirmed AHT. The absence of teachers was evident, as in the pre-lockdown group 40% (2) of referrals originated from schools compared to none during the lockdown period. Ophthalmic involvement was not present in any of the pre-lockdown cases and only 50% (2) of the during lockdown cases, with the appropriate proforma only used in one of these cases. Unfortunately, no further statistical testing was meaningful in light of the small sample size.

**Conclusions:**

The loss of the early warning detection mechanism provided by schools and health visitors may have contributed to both the change in presentation and severity of cases during the lockdown. There is also a need for ophthalmology and paediatrics to collaborate to ensure AHT cases are thoroughly investigated and documented.

## Background

Child abuse is an issue that manifests in all societies to some extent; the various types of abuse and their respective frequencies vary throughout the UK. It was estimated from the Crime Survey for England and Wales that one in five adults aged 18 to 74 years experienced at least one form of child abuse, whether emotional, physical or sexual, or witnessed domestic violence or abuse before the age of 16 years (8.5 million people) (ONS 2020).

Within the umbrella of physical abuse, the term *abusive head trauma* (AHT) is currently the accepted terminology that encompasses previously used terms such as non-accidental injury (NAI) or non-accidental head injury (NAHI) and shaken baby syndrome (SBS) (Watts 2013a). In the presence of head injury without any plausible medical explanation, the description of retinal findings helps distinguish between abusive damage and other causes. It is these ocular manifestations that ophthalmologists are vital in identifying and reporting (Bechtel et al. 2004; Watts 2013a). The presence of retinal haemorrhage (RH) was highly associated with definite or probable abuse versus definite or probable accident (Binenbaum et al. 2009).

Certain patterns of RH are more common in AHT, such as a large number of RH in both eyes, involving all layers of the retina, as well as extension into the periphery; however, there was no single retinal sign that was unique to abusive injury. RH is rare in accidental trauma and, when present, is predominantly unilateral (Maguire et al. 2013). RHs that are flame shaped and bilateral should be treated as suspicious of AHT in the absence of direct head trauma. The incidence of RH is convulsions (0.7%), chest compressions (0–2.3%), forceful vomiting (0%) and severe persistent coughing (0%). With these findings, only a trained ophthalmic specialist is able to detect AHT; therefore, and ophthalmic specialists can prove to be crucial in identifying victims of AHT.

When a new referral of possible AHT is identified, prompt involvement of the ophthalmic team is vital. It relies on good communication links between the relevant teams involved in the investigation, diagnosis and management of AHT.

The World Health Organization (WHO) declared COVID-19 a public health emergency on January 30, 2020, and on March 11, 2020, it was declared a pandemic and a global health emergency. The novel coronavirus (SARS-CoV-2) is a member of a large family of viruses called coronaviruses and was first described in the Wuhan province of China. It is a highly transmissible virus and is responsible for causing severe acute respiratory syndrome (SARS). The world was suddenly faced with a new set of challenging rules that included limiting our contact with others and the closure of non-essential businesses.

To mitigate the spread of the COVID-19, a ‘lockdown’ was introduced in England on March 23, 2020, and this continued in various forms until the first phased re-opening of schools in England on June 1, 2020. Lockdown measures included the closure of non-essential businesses, childcare facilities and schools. The government mandated social distancing measures in a bid to slow the spread, as it was evident that there was human-to-human transmission. The lockdown has had many foreseen and unforeseen consequences whose effects are still being understood in different fields; social, economic and health consequences are inevitable.

The lockdown has meant that families have had their support circles interrupted. Ultimately, this poses a risk, as telltale signs of abuse may go unnoticed. For this reason, AHT has sometimes been referred to as the ‘silent pandemic’ and become even more silent in the face of a global pandemic. Reduced exposure to services for children has caused widespread concern that child maltreatment may go undetected and that a surge of newly diagnosed victims of child abuse will emerge (Agrawal 2020; Rosenthal & Thompson, 2020; Seddighi et al., 2021; Teo & Griffiths 2020).

As a consequence of lockdown, children have spent an increased amount of time in the home, with less access to childcare facilities, decreased interactions with primary care health providers and a reduction in interaction with educators, who play a vital role in identifying abuse. In the United States, 20% of reports of abuse to protective services are made by educators (HHS 2013). The closure of schools for most pupils has been a critical point for families with children, especially those who are considered vulnerable. Definitions of vulnerability in children include children who are in care; children that care for others; children in households suffering domestic abuse, mental health problems, or drug or alcohol problems; children with special educational needs and disabilities (SEND); children who are outside mainstream education; and children on the edge of social care involvement, having been referred to social services in the last year but not meeting thresholds (Public Health England 2020).

The impact of lockdown and the changes to the way of life in many places of the world has been significant to both the psychological health and well-being of the population. It has been suggested in the UK that around 72% of people are worried about the effect of COVID-19 on their life, with high levels of anxiety in 32% of the population (ONS 2021). These adverse psychological effects of lockdown are multiplied due to other stressors resulting from the pandemic, such as unemployment (Brooks et al. 2020; Galea & Abdalla 2020).

Anecdotal evidence indicates there has been an increase in domestic violence during this period of lockdown (Bradbury-Jones & Isham 2020; Campbell 2020). Families confined to their homes without external support or contact with those that may be able to identify, help or signpost victims are unintentionally isolated without the usual scrutiny that may prevent this (Schols, de Ruiter & Öry 2013). It is postulated that the incidence of AHT in children may increase directly due to household isolation while detection may be decreased as children are hidden from healthcare professionals, teachers and social workers (Radford et al 2011). The pandemic may also increase AHT in children indirectly through the increased risk of domestic abuse during this time frame (Campbell 2020; Usher et al. 2020). In the United States, 60% of households where domestic abuse occurs contain children (Campbell et al. 2020). It has been estimated that children in households containing domestic violence are at a 60 times greater risk of child abuse than the general US paediatric population (Committee on Child Abuse and Neglect et al. 2010). The National Incidence Study in the United States estimated that 6% of children aged under 11 years, 19% of children aged 11–17 years, and 25% of those aged 18–24 years had experienced severe maltreatment at some point during childhood (Sedlak & Broadhurst 1996). Even in the pre-COVID-19 era, many child abuse cases may have been hidden, so these figures may be larger than estimated.

## Objectives

To investigate if there is a change in incidence and severity of abusive head trauma (AHT) in children between the initial three months of the first COVID-19 lockdown to the incidence and severity noted in the same time period in the preceding year. Of those cases, notes were reviewed to see which had ophthalmology input and how this was recorded.

## Data Source and Study Population

Data was collected from ***********, a major tertiary referral hospital, district general hospital and major trauma centre in **********, England, which forms part of the **********Trust.

Within the National Health Service (NHS), all staff who come into contact with children and their families have a responsibility to safeguard them when they have concerns; where appropriate, this means escalating to safeguarding teams within their respective areas. Data was made available for reported child protection referrals from the safeguarding team at ******* Hospital. Approval for the collection of data was obtained from the paediatric safeguarding and information governance lead for this study. We followed the Declaration of Helsinki and ethics committee principles. All data was collected on-site, and stayed on-site.

## Inclusion and Exclusion Criteria (Figure 1)

**Figure 1:**
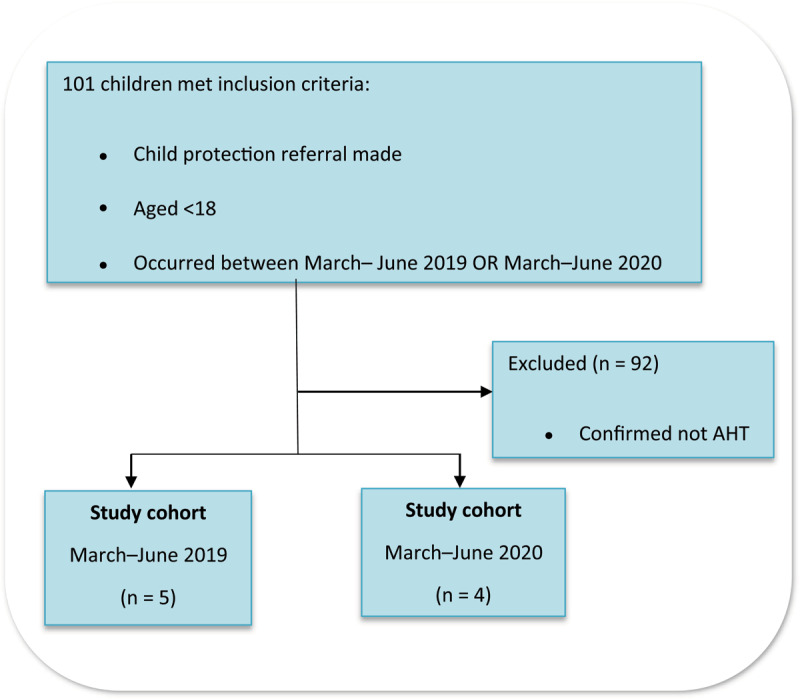
Flow diagram detailing the inclusion and exclusion of patients.

The participants for this study were selected using the following criteria:

Inclusion criteria:

- Child protection referral made
- Under 18 years old
- Occurred between March and June 2019 OR March and June 2020

Exclusion criteria:

- Patients confirmed not to have had any AHT (e.g., accidental, domestic, emotional, neglect, sexual).

## Data Collection

Cases with appropriate inclusion criteria and no exclusion criteria had data extracted from electronic medical records using a standardised data collection form by an ophthalmology registrar. Data was collected on demographic variables (age and gender), mode of presentation, date of presentation, whether the abuse was confirmed or suspected, systemic findings, skeletal imaging findings and whether an ophthalmic assessment was performed (defined as the entry into the clinical notes by an ophthalmic clinician); if so, the grade of the ophthalmic clinician, what was noted and whether a Royal College of Ophthalmologists proforma was used to record this were also collected (Watts 2013b). Anonymisation was performed on data collected for confidentiality.

We aimed to collect systemic findings, which we divided into facial bruising, non-facial bruising and conscious/unconscious. To assess whether the severity of injuries had been influenced by the lockdown period, we looked at previous studies that had attempted to predict the presence and severity of abuse based on bruise characteristics (Dunstan et al. 2002) and facial bruising in two different categories of severity and an additional informative category. Mild facial bruising was defined as a single bruise measuring <1cm^2^, and severe bruising was defined as multiple facial bruises or a single one measuring ≥1cm^2^. We also recorded whether any bruises could be classified as ‘patterned’ bruises (Kos & Shwayder 2006); these would include marks that indicated their origin, e.g., shape of the object used to inflict the bruise, slap or grab injuries in the shape of finger marks, ligature marks, etc., and could in turn inform regarding the severity of injuries inflicted. Additionally, as a consequence of the pandemic, it was thought that while social services would be aware of children with prior social service interactions, in light of the lack of published material on this, we would also record whether those children with confirmed AHT had previous involvement with social services or if they were new cases, previously unknown to social services.

## Statistical Analysis

Continuous variables are presented as a mean with standard deviation.

## Results

**Figure 2:**
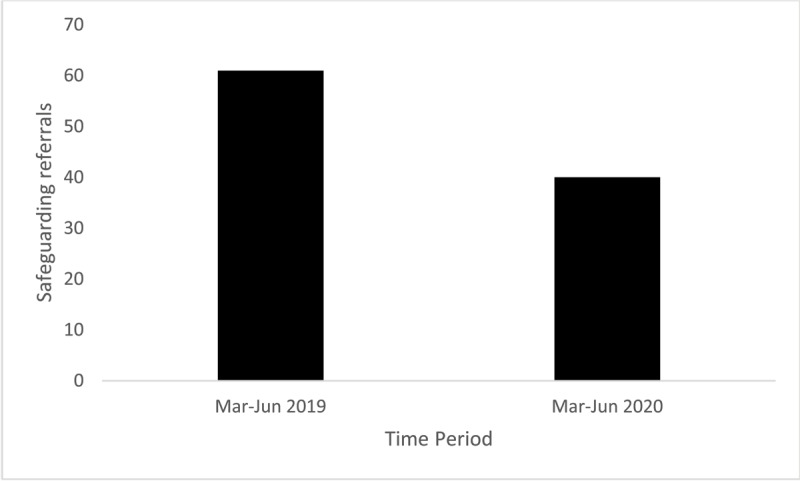
Safeguarding referrals made during pre-lockdown (March–June 2019) and during lockdown (March–June 2020).

Of the 101 safeguarding referrals reviewed, 60% (61/101) were pre-lockdown (March–June 2019) and 40% (40/101) during lockdown (March–June 2020). This represents a 34% reduction in safeguarding referrals made during the lockdown period (see Figure 2).

**Figure 3:**
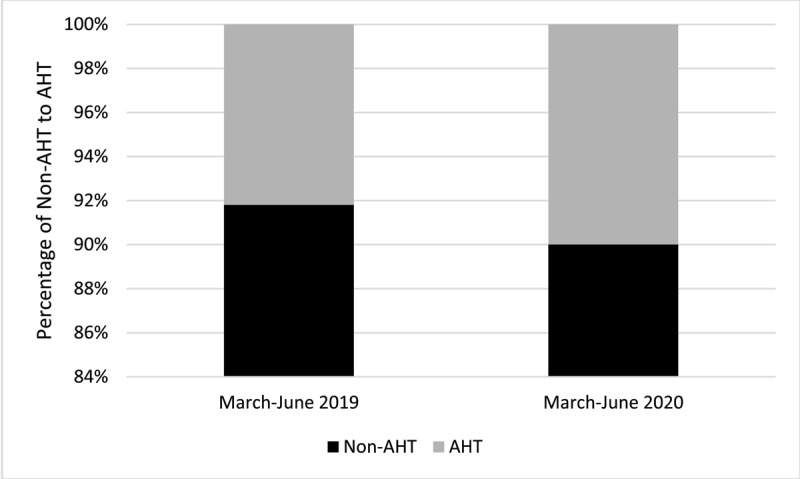
Zoomed-in bar chart showing the percentage of non-AHT compared to AHT during pre-lockdown (March–June 2019) and during lockdown (March–June 2020).

Of these referrals, 56/61 of the pre-lockdown safeguarding referrals were excluded as not being AHT, resulting in a pre-lockdown sample size of n = 5. Of the during lockdown group, 36/40 were excluded, resulting in a sample of n = 4 (Figure 3).

The incidence of AHT in the pre-lockdown and during lockdown group was 8.19% and 10%, respectively (see Figure 2): Risk ratio [RR], 1.22 [95% CI, 0.35–4.27] (Table 1).

**Table 1:** Table showing all safeguarding referrals received during the described time periods and those identified as AHT and non-AHT.


	*PRE-LOCKDOWN (MARCH–JUNE 2019)*	*DURING LOCKDOWN (MARCH–JUNE 2020)*

AHT	5	4

Non-AHT	56	36

Total	61	40

Cumulative incidence	8.19%	10%


The age of AHT cases pre-lockdown had a mean of 40 (±30.47) months and a median of 45 months. During the lockdown, AHT cases had a younger mean of 35.5 (±30.56) months and a median of 35.5 months. During the lockdown, more males were recorded suffering AHT (3:1) compared to pre-lockdown (3:2) (Table 2).

**Table 2:** Summary of patients with confirmed AHT.


	MARCH–JUNE 2019	MARCH–JUNE 2020

Number of patients (n)	5	4

Mean age (months)	40 (±30.47)	35.5 (±30.56)

Median age (months)	45	35.5

Age range (months)	1–76	3–68

Gender		

Males	3 (60%)	3 (75%)

Females	2 (40%)	1 (25%)


Modes of presentation displayed a shift in how cases were reported. The Accident and Emergency (A&E) Department and the police became the primary source of referrals during the lockdown period, 75% and 25%, respectively, compared to health visitors, schools and A&E in the pre-lockdown period, 40%, 40%, 20%, respectively (Table 3). It was also noted that of those referrals identified as being AHT, the proportion previously known to social services pre-lockdown and during lockdown were 3 (60%) and 1 (25%), respectively.

**Table 3:** Demonstrating where safeguarding referrals were initially generated from.


MODE OF PRESENTATION	MARCH–JUNE 2019	MARCH–JUNE 2020

**Accident & Emergency Dept**.	1 (20%)	3 (75%)

**Police**	0	1 (25%)

**Health visitor**	2 (40%)	0

**School**	2 (40%)	0


It is not possible to produce further meaningful statistical analysis comparing the incidence of AHT between the two time periods due to the small sample size precluding either the Pearson chi-square test or a meaningful result from a two-sided Fisher’s exact test. There is, therefore, not enough evidence to reject H_0_ in favour of H_1_.

The findings noted during the pre-lockdown period showed that all cases involved mild facial bruising (Table 4). This contrasts with the during lockdown period, where equal proportions of mild and severe facial bruising recorded at 50% (2) and 50% (2), respectively. There were no cases of patterned bruising recorded. The only episode of unconsciousness at presentation was noted during the lockdown period.

**Table 4:** Paediatric systemic findings. Presenting signs in children confirmed with AHT.


	MARCH–JUNE 2019	MARCH–JUNE 2020

**2020 Facial bruising**	5 (100%)	4 (100%)

Mild facial bruising	5 (100%)	2 (50%)

Severe facial bruising	0	2 (50%)

Patterned bruising	0	0

**Non-facial bruising**	0	0

**Conscious**	5 (100%)	3 (75%)

**Unconscious**	0	1 (25%)


Of the nine patients in this cohort, 89% (8) had facial bruising, and 11% (1) patient had ear bruising. Bruising in obvious areas on the face/head was the reason these children were brought to the attention of medical professionals. This highlights the importance of looking out for children who are possibly suffering from physical abuse but have no obvious physical signs of it in easy-to-see areas such as the face. In the pre-lockdown group, 80% (4) were brought to the attention of professionals by social services/health visitors. The absence of this visibility to multi-disciplinary professionals could mean that some children suffering from physical abuse were possibly undetected during lockdown. This further reinforces the need for identification of vulnerable and ‘at-risk’ children in particular in the event of another lockdown or unplanned interruption to schooling.

In the pre-lockdown period, an MRI head scan was performed on one child compared to CT head imaging performed on 50% (2) of children during the lockdown period. None of the during lockdown group underwent an MRI scan.

This study showed that of the nine patients in the study, only 33% (3) patients had any form of imaging performed. In the pre-lockdown group 20% (1) had a CT scan, and the other 80% (4) had no imaging performed. Of note, 50% (2) had significant facial bruising in the during lockdown group, and only one of these, who presented unconscious, underwent imaging.

Of the pre-lockdown group 40% (2) were under the age of one year, and of these, only 50% (1) had imaging in the form of MRI scan performed. In the during lockdown group, 50% (2) were under the age of one year. Of these, only 25% (1) child had imaging performed (Table 5).

**Table 5:** Documented investigations in children confirmed with AHT.


IMAGING MODALITY	MARCH–JUNE 2019	MARCH–JUNE 2020

**MRI head**	1 (20%)	0

**CT head**	0	2 (50%)

**Ophthalmic assessment**		

**Performed**	0	2 (50%)

**Not performed**	5 (100%)	2 (50%)

**Grade of ophthalmologist**		

**ST7**	0	1 (50%)

**Consultant**	0	1 (50%)

**Ophthalmology proforma used**		

**Used**	N/A	1 (50%)

**Not used**	N/A	1 (50%)


Ophthalmic examination was not performed on any patients presenting during the pre-lockdown period, whereas it was performed on 50% (2) of children during the lockdown period. Of the ophthalmic assessments performed, 50% (1) were performed by a senior trainee (ST7) and a consultant ophthalmologist, one of which used the ophthalmology AHT proforma.

In the during lockdown group, only 25% (1) had ophthalmic findings. Of the pre-lockdown group, there were no ophthalmic findings, as 0% had ophthalmic involvement. A limitation of this study is that we cannot be sure that all children who presented with confirmed or suspected AHT were referred for ophthalmology input. It is possible that if the ophthalmology team had been involved in every case of suspected or confirmed AHT, we may have found a higher incidence of ophthalmic findings.

Unfortunately, it was again impossible to produce meaningful statistical analysis comparing the severity of the pre-lockdown and during lockdown periods due to the small sample sizes precluding either the Pearson chi-square test or a meaningful result from a two-sided Fisher’s exact test.

## Discussion

Our study found no significant change in the incidence or severity of abusive head trauma (AHT) in the paediatric population pre- and during the COVID-19 lockdown in the North East of England. The during lockdown group found bilateral retinal haemorrhage in 25% (1) of patients compared to no ophthalmic findings in the pre-lockdown group. This was due to no ophthalmic involvement in all the pre-lockdown cases. This is an important finding, as it shows the lack of engagement and/or awareness of the importance of ophthalmic involvement in such cases.

Most countries widely employed social distancing and movement restriction in the form of lockdowns during the pandemic in an attempt to control the reproduction number of the coronavirus, buying time for health care systems to cope with surges in demand as well as allowing for the production of vaccines. As with all interventions used on such a large scale, unrecognised and unintended consequences may result, and as such, the risk of child abuse and AHT has been suggested to increase during this period (Peterman et al. 2020). Similar patterns in child abuse frequency have been reported around the periods when children spent more time at home, such as during summer holidays (Leaman, Hennrikus & Nasreddine 2017).

It was important to note that more referrals were made to safeguarding services during the pre-lockdown period, which likely reflects the greater visibility children have to multi-disciplinary agency members such as health visitors and teachers during a fully functioning non-locked-down society. Teachers have a vital role in the detection and reporting of child abuse, as they encounter almost all children in the population through their role (Schols, de Ruiter & Öry 2013). Table 3 demonstrates the loss of referrals from teachers, and it is the authors’ and our colleagues’ suspicion that the loss of this early warning detection mechanism may have contributed to the change in presentation and severity. This requires further research to identify methods of preventing this should further lockdowns or interruptions to children’s education occur in the future. There is a concern that as lockdown is relaxed, there may be an increase in cases of recognised child abuse, which were undetected during lockdown (Agrawal 2020; Kovler et al. 2021).

In a lockdown, it could be expected that the associated loss of childcare arrangements, economic uncertainty (Berger et al. 2011), social isolation and the consequences on parents’ and carers’ mental health that there would be a correlated increase in frequency and severity of AHT (Conrad-Hiebner & Byram 2020). Although our small sample cannot definitively show any statistically significant changes that let us reject H_0_, it highlights to us the fact that children have had a loss of visibility to multiple social mechanisms that might recognise and report risk factors or evidence of abuse. This is also highlighted by the lower levels of presentation to A&E pre-lockdown, which suggests that children may have been identified as possibly suffering from physical abuse/AHT by multi-disciplinary professionals that had seen the children and consequently raised the alarm, so to speak, and sought medical help. The absence of this avenue of visibility for children during lockdown may have contributed to the higher rate of presentation to A&E.

It is important to consider the limitations of this study. This retrospective study is severely limited by the short period, small catchment area, and the associated small number of patients. Additionally, we were only able to review children who had safeguarding referrals performed and therefore did not capture cases of AHT that did not come to the attention of authorities. The exclusion criteria for patients classified as not AHT is a possible source of false negatives if patients were erroneously classified as accidental rather than abusive. Our patients were derived from a specific geographical area in the UK which may not be generalisable to other sites and may be specific to hitherto unknown socio-economic factors affecting adversity in the area.

Not all families may have been adversely affected by the COVID-19 lockdown. Furlough schemes were provided by the UK government under which firms could ‘furlough’ employees and apply to the government for a grant that would cover a portion of each worker’s usual monthly wage. Previous studies have shown that the mental health decline associated with AHT (Aldridge 2006) is ameliorated significantly by employment protection (Barlow et al. 2019; Cook, Giovanis & Gobey 2020). This may result in some parents having reduced stressors compared to normal, decreasing the risk of AHT.

Out of the nine recognised cases of AHT, only two had an ophthalmic assessment, of which one was accompanied by a corresponding proforma. A child suspected of physical abuse should be assessed from an ophthalmic point of view as part of a multi-disciplinary assessment. The Royal College of Ophthalmologists (RCOphth) and the Royal College of Paediatrics and Child Health (RCPCH) have created guidance on the evaluation of a child with suspected head trauma that states that a child suspected of AHT should be referred to the ophthalmologist for clinical assessment. The joint working group has designed a standardised clinical proforma for documentation. It has been clinically established that retinal haemorrhages have a high positive predictive rate for AHT (Watts 2013). In our study, of the nine patients that were included, one patient had subdural haemorrhage, and the same patient was confirmed to have bilateral retinal haemorrhages. The low number of patients with ophthalmic involvement shows that a better understanding and more robust communication system is needed amongst paediatricians and ophthalmologists. We also need to prioritise auditing our protocols against the national guidelines for continuous improvement.

The Royal College of Radiologists has created recommendations to assist clinicians in decision making concerning suspected physical abuse:

- Imaging should always include skeletal survey in children under two years old and skeletal survey and CT head scan in children under one year old.
- Children who are older than one year and have external evidence of head trauma and/or abnormal neurological symptoms or signs should also have a CT head scan.
- Skeletal survey may occasionally be indicated in older children, and this should be considered on a case-by-case basis (Royal College of Radiologists and Society and College of Radiographers 2018).

In view of the above recommendations, it was difficult to decipher from the medical notes that were available whether a skeletal survey was done in the patients who fell within the guidance set above. This reinforces the importance of being aware of and following guidance set by the RCOphth and other professional bodies, such as the Royal College of Radiologists. Further collaborative work is required in this area to work on improving communication and awareness of recommendations, documentation and auditing to ensure we are following the latest guidelines and achieving the best outcomes for these patients.

Of note, there were no episodes of patterned or non-facial bruising reported in either group reviewed, whether this is because a certain type or severity of abuse is required to generate these findings or they were not identified is unclear. However, there is a case to ensure clinicians are aware to regularly look out for and document this type of injury.

The study was retrospective, performed remotely on previously collected data. It was therefore not able to capture the views of children or their carers regarding the impact that lockdown has had on them. Further research into this area is required as lockdowns end to identify the groups most affected by this to allow delivery of focused care and intervention and recognition on how to prevent this should any future interruptions to schooling ever occur. This could involve facilitating all stakeholders’ input on the issue and allowing more evidence to help guide future prevention and intervention programs.

## Conclusions

Our study found no significant change in the incidence or severity of abusive head trauma (AHT) in the paediatric population pre- and during COVID-19 lockdown in the North East of England. The during lockdown group found bilateral retinal haemorrhage in one (25%) patient compared to no ophthalmic findings in the pre-lockdown group. This was due to no ophthalmic involvement in all the pre-lockdown cases. This is an important finding, as it shows the lack of engagement and/or awareness of the importance of ophthalmic involvement in such cases.

This study adds to the growing body of evidence that as children have come out of lockdowns and returned to school in the UK, we should be vigilant for previously unreported incidents of AHT.

Although our data has a limited sample size, it has highlighted the loss of exposure children have to both teachers and health visitors as a consequence of the lockdown. As we have discussed, they form a key mechanism to identify children in need of support. Our data also demonstrates that there may be a wider trend warranting further study, looking at the recovery from lockdowns, the resultant cases that come to light and the severity of both AHT and other types of abuse. The majority of children presented via A&E during lockdown (75%) compared to pre-lockdown (20%). This highlights that the visibility of children from other multi-disciplinary professional agencies outside of the home plays an important safety net for children who may be suffering from or are at risk of physical abuse.

We should be aware that regardless of the broader social issues that may occur as a result of the COVID-19 pandemic, there is an enduring need for multi-level stakeholder cooperation to protect and ensure children’s safety (Sserwanja, Kawuki & Kim 2021). This may require attempting to catch up, so to speak, with the opportunities missed during lockdown by identifying those most vulnerable and being vigilant for any future signs of abuse despite stretched health and social care budgets.

There were inconsistencies in which patients had an ophthalmology review, and there were inconsistencies regarding which child underwent imaging. This study identifies the need for a rigorous work-up of a child who presents with suspected or confirmed AHT from the involved specialities in a logical and timely manner.

This study also highlights that any ophthalmic clinician involved in assessing a child for possible AHT should complete the Royal College of Ophthalmologists proforma following an ophthalmic examination. In our study, only one out of the nine reviews had a completed proforma. Completion of this form would provide useful data for future audits and enable us to work collaboratively with other trusts to build stronger research projects.

Our study shows a need for not only more research to identify specific at-risk groups and effective interventions but also multi-level stakeholder cooperation to ensure increased funding, increased community awareness and sensitisation, early detection and effective management and referral of child abuse cases in the recovery from lockdowns and for any future prolonged absences from school for children.
